# Heats of Hydrolysis and Formation of Dimethoxychloroborane

**DOI:** 10.6028/jres.065A.045

**Published:** 1961-10-01

**Authors:** Marthada V. Kilday, Walter H. Johnson, Edward J. Prosen

## Abstract

The heat of hydrolysis of dimethoxychloroborane has been measured; for the reaction,

$$\begin{array}{c} {\left({\text{CH}}_{3}O\right)}_{2}\text{BCl}(\text{liq})+3H_{2}O(\text{liq})=H_{3}{\text{BO}}_{3}(c)+2{\text{CH}}_{3}\text{OH}(\text{liq})+\text{HCl}(g)\\ \Delta H(25{}^{\circ}C)=-26.6\pm 0.8\;\text{kj}/\text{mole}\\ \;=-6.4\pm 0.2\;\text{kcal}/\text{mole}. \end{array}$$

From this, we have calculated the heat of formation of dimethoxychloroborane: for the liquid, Δ*Hf*° (25 °C) = −782.1 ± 1.8 kj/mole (−186.9±0.4 kcal/mole), and for the gas, Δ*Hf*° (25 °C) = −747.9 ±2.2 kj/mole (−178.8±0.5 kcal/mole).

## 1. Introduction

Heats of formation for the three ethoxy derivatives of boron trichloride have been determined [1, 2];^1^ however, in the series of methoxy derivatives, the heat of formation of only trimethoxyborane (or methyl borate) has been reported [1]. As part of the program at the National Bureau of Standards for determining thermochemical properties of boron-containing compounds, we have measured the heat of hydrolysis of liquid dimethoxychloroborane. From this, we have calculated values for the heats of formation of liquid and of gaseous dimethoxychloroborane; we have also estimated a value for the heat of formation of gaseous methoxydichloroborane.

## 2. Materials and Apparatus

The sample of dimethoxychloroborane (DMCB) was purified by fractional crystallization.^2^ Freezing-point measurements indicated a purity of about 95 percent DMCB.^2^

Trimethoxyborane (or methyl borate) is believed to be the principal impurity in the sample for three reasons. First, its boiling point, 68.7 °C [3], is near that of dimethoxychloroborane, 74.7 °C [3], and the two might distill simultaneously. The boiling point of methoxydichloroborane, 58 °C, is sufficiently removed that distillation should provide a satisfactory separation. Second, the sample was probably the product of reaction between boron trichloride and methyl alcohol where methoxy substitution for one, two, or three chlorine atoms occurs depending on the mole ratios of the reactants [3]; therefore, methyl borate might be present in small amounts. The third reason is that titrations of the product of hydrolysis of the sample in water indicate an average of 99.5 percent by weight of the boron and only 92.6 percent by weight of the chlorine expected if the sample were pure DMCB. Thus, the impurity contained approximately the same amount of boron per gram as DMCB and essentially no chlorine; this is true of methyl borate. If the 5 mole percent impurity found from freezing-point measurements were methyl borate, the analysis would be 100.2 percent of boron and 95.2 percent of chlorine.

Further information about the composition of the impurity was sought and two methods for determining carbon in the sample were employed. In the first method, a DMCB sample was burned in an oxygen bomb; the CO_2_ in the combustion products was absorbed by Ascarite and weighed. In the second method, a C-H ratio was determined from combustion in a tube furnace.^3^ Unfortunately, the results of both methods were inconclusive because of the limitations and uncertainties imposed by the presence of chlorine and boron in the sample, and by the fact that the amount of impurity was small.

The sample used in determining the heat of solution of crystalline boric acid was obtained by the recrystallization from aqueous solution of analytical-grade boric acid. The sample was air-dried at room temperature and placed in spherical Pyrex-glass bulbs which were then evacuated at approximately 10^−5^ mm of Hg pressure and room temperature for 6 hr to remove surface moisture before sealing. It is interesting to note that this long exposure to reduced pressure apparently did not cause decomposition of the H_3_BO_3_, since the ratios of the weight of H_3_BO_3_ titrated in the final calorimetric solutions to the weight of sample were 0.997, 1.005, 0.993, and 0.997.

Glass ampoules are generally desirable sample containers in solution calorimetry, but they were unsatisfactory for the DMCB samples because the reaction became so violent upon breaking the ampoule that the calorimetric solution was spattered and the gaseous products were lost before being dissolved. Even when only the capillary tip of the ampoule was broken, enough water was drawn into the bulb by the partial vacuum to produce an explosion.

A satisfactory reaction rate was achieved when the sample was enclosed in a monel cylinder (capsule) with a diaphragm of Mylar^4^ film closing both ends. The reaction started when the film was pierced with a nichrome needle.

**Figure 1:**
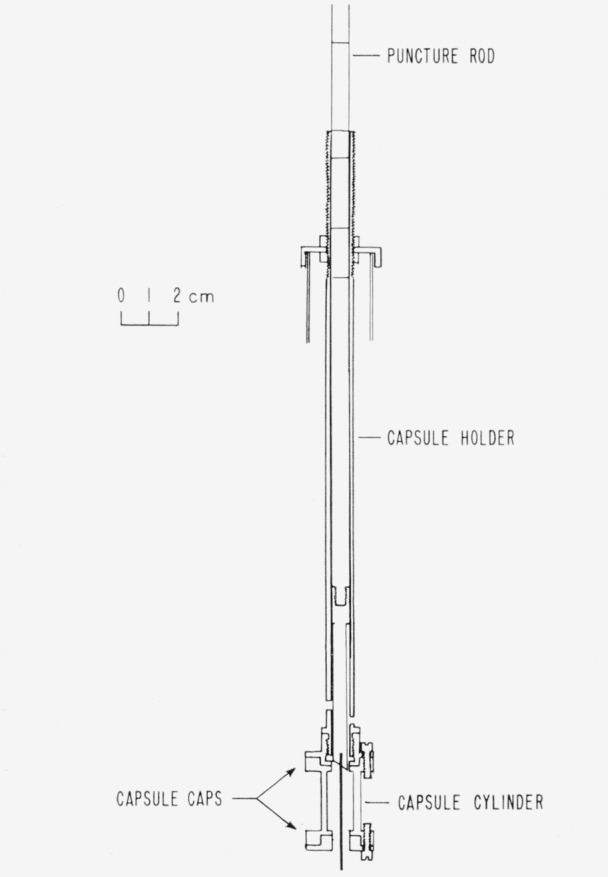
Diagram of sample holder.

The capsule which contained the samples during calorimetric experiments is shown in figure 1. The capsule, capsule-holder, and lower end of the puncture-rod were made of Monel to resist attack by hydrochloric acid formed in the reaction of the DMCB with water. The upper end of the puncture-rod was made of polystyrene to minimize heat leakage from the calorimeter. The lower end of the Monel rod was cut at an angle of 30° and the edge was sharp for cutting the Mylar diaphragms. A nichrome needle, 1-mm diam, was attached at the center of the lower end of the Monel rod; vertical grooves in the rod permitted the displacement of liquid as the rod entered the capsule. The Monel plate, or cap, at each end of the cylindrical capsule was held in place by three Monel screws. A Mylar diaphragm, 0.025-mm thickness, and a polyethylene gasket, 0.13-mm thickness, closed the cylinder at each end; the cylinder was sealed when the screws were tightened. The lower end of the capsuleholder was threaded so that the capsule could be attached to it easily and securely; several holes in the walls of the holder permitted circulation of the calorimetric solution.

**Figure 2:**
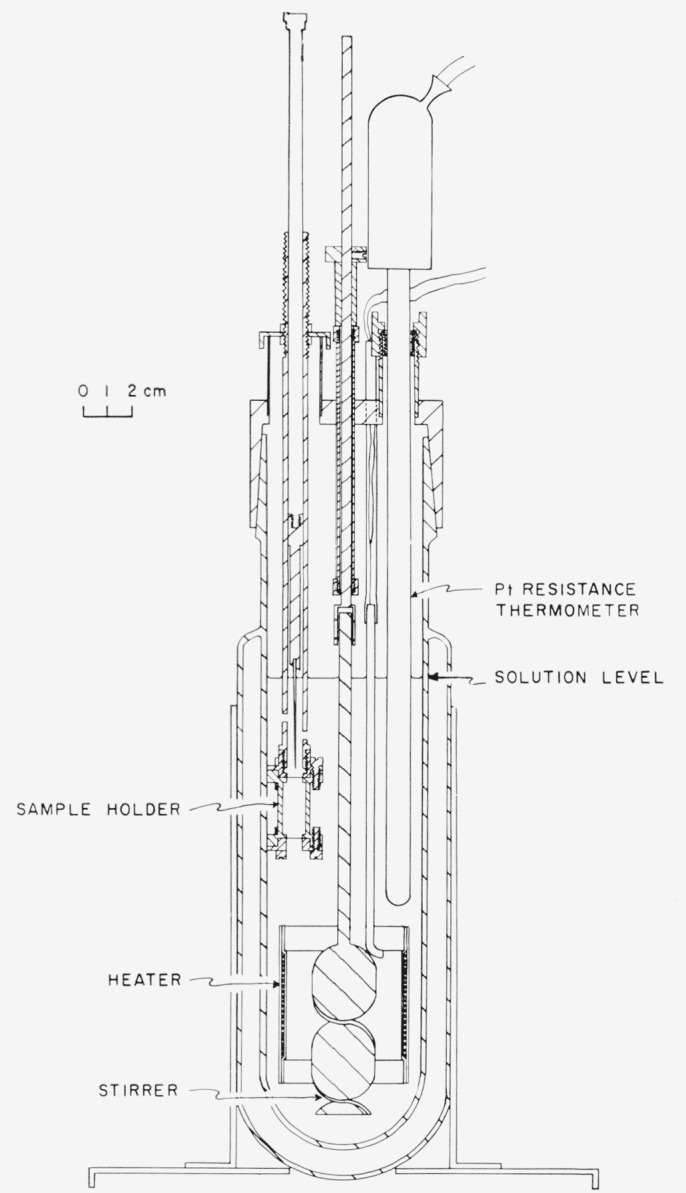
Diagram of solution calorimeter.

The solution calorimeter is shown in figure 2. The body of the vessel is Pyrex glass with a standard-taper, ground-glass joint at the top and a silvered vacuum-jacket. The cap is nickel-plated brass with gas-tight fittings for a stirrer, a platinum resistance thermometer, a capsule-holder, two heater-leads, and a gas exit-tube (not shown). The stirrer-support and shaft are stainless steel with two Teflon bearings, and the portion of the stirrer in contact with the solution is Pyrex glass. The stirrer was driven by a motor at approximately 500 rpm.

The brass case for the calorimeter heater consisted of an inner cylinder with ⅛-inch tubes for leads welded inside on opposite sides of the cylinder (only one lead-tube is shown), and an outer-cylinder which was a tight fit over the inner cylinder; the walls of the case are approximately ¼-mm thickness. The lacquered manganin heater wire, No. 36 B and S gage, of 121-ohm resistance was non-inductively wound over a thin sheet of mica covering the depression on the inner cylinder, and a second sheet of mica was placed between the wire and the outer cylinder. The two rims of the case were soft-soldered and flashed with copper; then the entire assembly, including the two lead-tubes, was plated with gold to a minimum thickness of 0.08 mm. Above the solution level the lead-tubes were attached to glass tubing with a DeKhotinsky cement. A pair of copper leads, for current and potential measurements, extended from each lead-tube.

The calorimeter was immersed in a water bath in which the temperature, approximately 26.8 °C, varied by not more than ±0.002 °C during an experiment. The bath, temperature controls, and method of measuring electrical energy during calibrations have been described previously [4,5].

Measurements of the current and voltage across the heater were made alternately with a Wenner potentiometer at 2-min intervals; i.e., the current was measured on the first minute, the voltage on the second, etc. Temperature measurements were made with a calibrated platinum resistance thermometer which was enclosed in a glass sheath and had a resistance at 0 °C of 25.473 ohms; the resistance was measured with a Leeds and Northrup G-2 bridge in conjunction with a high-sensitivity galvanometer.

## 3. Experimental Procedures

Portions, weighing 1.0 to 1.5 g, of the DMCB sample were distilled under vacuum into glass ampoules which were then sealed and stored at −15 °C. These ampoules were made of Pyrex-glass tubing, 7-mm diam and 100-mm long, with a scratch around the middle to facilitate breaking later. The sample occupied about one-third of the total volume of the ampoule.

Before a calorimetric experiment, a sample was transferred from a glass ampoule to the Monel capsule (fig. 1). All parts of the capsule including the diaphragms and gaskets were carefully weighed before placing them in a dry box containing a helium atmosphere.^5^ The top gasket, diaphragm, cap, and screws were assembled; then the capsule was inverted and screwed onto a support which facilitated subsequent manipulations. The glass ampoule containing the sample was broken at the scratch, and the sample was poured through a funnel into the cylinder. The second diaphragm, gasket, and cap were assembled, and the screws were tightened sealing the capsule.

A minimum time elapsed between removing the capsule from the dry box and weighing it, to reduce errors resulting from exchange of helium and air through the Mylar diaphragms. When the capsule contained a sample of DMCB, there was a gain in weight of about 0.2 mg during the first hour, and 0.1 mg or less during the second hour; the balance case was saturated with water vapor at 25 °C. However, when the capsule contained liquid water, and the atmosphere of the balance was dried by anhydrous magnesium perchlorate, no change in weight was observed during a period of more than 2 hr. Therefore, we concluded that the Mylar diaphragms were, for the requirements of this work, impervious to moisture. The gain in weight observed when the capsule contained the sample, was apparently the result of exchanging helium with air and of adsorption of moisture on the capsule after removing it from the dry box. The weighing errors involved were less than 0.1 percent of the sample weight.

For the hydrolysis of the DMCB the calorimeter contained 450 g of distilled water (25 moles). An electrical calibration preceded each chemical reaction. Each experiment included a 20-min equilibration period, a 20-min initial rating period, a 20-min calibration period which included 9 min of electrical heating, a 20-min middle rating period, a 20-min chemical reaction period, and a 20-min final rating period. Temperatures were recorded at 2-min intervals during the three rating periods, and at 1-min intervals during the calibration and chemical reaction periods.

Five marks on the upper end of the puncture-rod (fig. 1) indicated various fixed positions used during the calorimetric experiments. The lowest mark was at the initial position where the needle was not in contact with the capsule. To start the reaction, only the top diaphragm was pierced with the nichrome needle. After 1 min the puncture-rod was pushed down to the point where the needle punctured the bottom diaphragm. On the third minute the rod was withdrawn to the initial position, and the following minute both diaphragms were again pierced and the needle again withdrawn to the initial position. On the fifth minute the top diaphragm was cut out by the sharp edges on the Monel puncture-rod; the bottom diaphragm was cut out on the sixth minute. Thus, when the rod was returned to its initial position, the calorimetric solution was free to circulate through the capsule. Most of the reaction occurred as the bottom diaphragm was first punctured with the needle, but the reactions were never as violent as when the samples were contained in glass ampoules.

The final calorimetric solutions were titrated with 0.1*N* sodium hydroxide solution to determine the amounts of hydrochloric acid and of boric acid. End points were taken from curves obtained by plotting the volume of standard alkali solution versus *p*H as measured on a Beckmann *p*H meter. The first end point, occurring at *p*H 5.6, indicated the volume of sodium hydroxide equivalent to the HCl. d-Mannitol was then added to the solution forming a complex with the boric acid and increasing its acidity; a second titration curve was obtained with an end point at *p*H 7.6 which indicated the volume of alkali equivalent to the boric acid.

## 4. Data and Discussion

Results of the dimethoxychloroborane (DMCB) hydrolysis experiments are given in table 1. *E*_a_ is the energy equivalent of the initial system as determined in an electrical calibration which preceded the chemical reaction. Δ*Rc* is the temperature rise measured during the chemical reaction period and corrected for cooling and stirring energies as described by Prosen [6]. In the 4th column are the weights *in vacuo* of the DMCB samples; the buoyancy factor was calculated using 1.2 g/ml as the density of the samples. This density is an approximation obtained from weights of samples contained in spherical glass bulbs of known volumes; it is believed to be within 10 percent of the correct value.

The number of moles of HCl and of H_3_BO_3_ given in the table were obtained from the titrations of the DMCB hydrolysis solutions. In DMCB the chlorine and boron are equiatomic, but the titrations indicated about 7 mole percent less HCl than H_3_BO_3_. To eliminate loss of chlorine by evaporation as a possible cause for the low value obtained in the titrations, the following experiment was performed. A sample of DMCB in a thin glass bulb, 150 ml of water, and a monel weight for crushing the bulb were placed in a bomb ordinarily used for oxygen combustions. The bomb was tightly closed, then shaken vigorously to break the bulb and to dissolve the gaseous products in the water. The resulting solution was titrated immediately. Two such experiments yielded 0.0212 mole HCl and 0.0226 mole H_3_BO_3_, and 0.0162 mole HCl and 0.0175 mole H_3_BO_3_, respectively; the HCl was 6 and 7 mole percent less than the H_3_BO_3_. Therefore, the chlorine deficiency in the products of hydrolysis was assumed to result from an impurity in the DMCB sample.

As mentioned previously, we have reason to suspect that the principal impurity was methyl borate. It fulfills the above requirements of a lower chlorine content than DMCB, and almost the same molecular weight, and has a slightly lower boiling point than DMCB. There is some evidence of fractionation of the sample as may be seen by inspection of table 1. The Sample Number indicates the order in which the calorimetric samples were distilled from the main sample. (Samples 4 and 5 were two small samples which were combined for one calorimetric experiment.) The weight percents given in the table are the ratios of the weight of the element found in titration to the weight of the sample. The weight percent of chlorine tends to increase with each sample while the weight percent of boron remains constant; this we would expect as a greater proportion of the more volatile impurity is transferred in the first portions distilled.

Reduction of the data from the calorimetric experiments given in table 1 was made with the following assumptions:

1. Methyl borate was the only impurity in the samples.
2. The number of moles of methyl borate was equal to the difference between the number of moles of HCl and the number of moles H_3_BO_3_ titrated in the hydrolysis solutions.
3. The number of moles of DMCB was equal to the number of moles of HCl found by titration of the hydrolysis solutions.

For conversion to the thermochemical calorie the relation, 1 thermochemical calorie = 4.1840 joules was used. The atomic weights used are from the 1957 International Table of Atomic Weights [7].

The heat contributed by the methyl borate impurity, *q*_MB_, is the product of the number of moles of methyl borate and the heat of hydrolysis of methyl borate, 18.01 kj/mole, measured by Charnley, Skinner, and Smith [1]. The following equation was used to obtain the heats of reaction:

$$\Delta H(26.8{}^{\circ}C)=\frac{E_{a}(\Delta Rc)-q_{\text{MB}}}{\text{HCl titrated}\times 1000}.$$

No correction was made for the energy required to break the Mylar diaphragms because it was found to be of the order of 0.2 joule which was near the limit of detection.

The mean value for the heat of reaction, ΔH(26.8 °C) = −94.77 kj/mole or −22.65 kcal/mole, has been converted to 25 °C by use of Δ*Cp*=51 cal/mole °C obtained from the following estimated heat capacity and apparent molal heat capacities: (CH_3_O)_2_BCl (liq), 40 cal/mole °C; H_3_BO_3_ in final solution, 30 cal/mole °C; HCl in final solution, −27 cal/mole °C; and CH_3_OH in final solution, 20 cal/mole °C. Thus for the reaction,

$$\begin{array}{c} {\left({\text{CH}}_{3}O\right)}_{2}\text{BCl}(\text{liq})+3H_{2}O(\text{liq})=[H_{3}{\text{BO}}_{3}+\text{HCl}+2{\text{CH}}_{3}\text{OH}](3000H_{2}O)\\ \Delta H(25{}^{\circ}C)=-94.39\pm 0.75\;\text{kj}/\text{mole}\\ \;=-22.56\pm 0.18\;\text{kcal}/\text{mole}. \end{array}$$ (1)

The uncertainty of about 1 percent was assigned to this value to include the experimental reproducibility and analytical errors as well as the uncertainties introduced by unidentified impurities and possible polymerization products.

To derive the heat of formation of DMCB from the heat of hydrolysis, it is necessary to know the heats of formation of CH_3_OH, H_3_BO_3_, H_2_O, and HCl and to employ the heats of three auxiliary reactions: (1) the heat of solution of crystalline boric acid, (2) the heat of solution of HCl gas in boric acid solution, and (3) the heat of solution of methyl alcohol in the solution of boric acid and hydrochloric acid.

The heat of solution of crystalline boric acid has been measured in several laboratories [8, 9, 10, 11, 12]; as there is disagreement in the results reported, we made four determinations at 25 °C. The data for these experiments are given in table 2, where the number of moles of H_3_BO_3_ was calculated from the titration of the final solutions. Thus we obtain for the reaction:

$$\begin{array}{c} H_{3}{\text{BO}}_{3}(c)=H_{3}{\text{BO}}_{3}(1900H_{2}O)\\ \;\Delta H(25{}^{\circ}C)=+21.94\pm 0.16\;\text{kj}/\text{mole}\\ \;=+5.24\pm 0.04\;\text{kcal}/\text{mole}. \end{array}$$ (2)

^6^

The uncertainty is taken as twice the standard deviation of the mean of the experimental values.

The heat of solution of gaseous hydrochloric acid in boric acid solution was not determined. However, the heat of dilution of concentrated hydrochloric acid (37.39% HCl) in 0.014*M* H_3_BO_3_ solution was measured in four experiments. The result was the same as for comparable dilution in water. We, therefore, assumed that the heat of solution of gaseous hydrochloric acid in 0.019M H_3_BO_3_ solution is equal to the heat of solution of gaseous hydrochloric acid in water [13] as follows:

$$\begin{array}{c} \text{HCl}(g)+H_{3}{\text{BO}}_{3}(3000H_{2}O)=[\text{HCl}+H_{3}{\text{BO}}_{3}](3000H_{2}O)\\ \Delta H(25{}^{\circ}C)=-17.95\;\text{kcal}/\text{mole}. \end{array}$$ (3)

The heat of solution of methyl alcohol in a solution 0.014*M* in boric acid and 0.012*M* in HCl was also measured and found to be equal to the heat of solution of methyl alcohol in water at infinite dilution [13]. Thus, we assume

$$\begin{array}{c} 2{\text{CH}}_{3}\text{OH}(\text{liq})+[H_{3}{\text{BO}}_{3}+\text{HCl}](3000H_{2}O)=\\ \left[H_{3}{\text{BO}}_{3}+\text{HCl}+2{\text{CH}}_{3}\text{OH}](3000H_{2}O\right)\\ \Delta H{}^{\circ}(25{}^{\circ}C)=-3.50\;\text{kcal}/\text{mole}. \end{array}$$ (4)

By subtracting the sum of eqs (2, 3, and 4) from eq (1), we obtain

$$\begin{array}{c} {\left({\text{CH}}_{3}O\right)}_{2}\text{BCl}(\text{liq})+3H_{2}O(\text{liq})=H_{3}{\text{BO}}_{3}(c)+\\ 2{\text{CH}}_{3}\text{OH}(\text{liq})+\text{HCl}(g)\\ \Delta H{}^{\circ}(25{}^{\circ}C)=-26.57\pm 0.79\;\text{kj}/\text{mole}\\ \;=-6.35\pm 0.19\;\text{kcal}/\text{mole}. \end{array}$$ (5)

The uncertainty assigned to this value is taken as the square root of the sum of the squares of the individual uncertainties involved in the calculations. Estimated uncertainties of ±0.02 kcal/mole were assigned to reactions 3 and 4.

To calculate the heat of formation of DMCB we used for crystalline boric acid, Δ*Hf*° (25 °C) = −262.16 kcal/mole derived from the value for the heat of formation of boric oxide based on crystalline boron obtained by Prosen, Johnson, and Pergiel [4]. The heats of formation at 25 °C of the other compounds were taken from [13]: CH_3_OH(liq), −57.02 kcal/mole; HCl(g), −22.019 kcal/mole; and H_2_O (liq), −68.317 kcal/mole. Combining these with the heat of reaction (5) we obtained for the heat of formation of liquid dimethoxychloroborane

$$\begin{array}{c} \Delta Hf{}^{\circ}(25{}^{\circ}C)=-782.07\pm 1.84\;\text{kj}/\text{mole}\\ \;=-186.92\pm 0.44\;\text{kcal}/\text{mole}. \end{array}$$

The uncertainties considered here include that of reaction (5), ±0.32 kcal/mole for the boric acid, ±0.1 kcal/mole for the methyl alcohol, ±0.02 kcal/mole for the hydrochloric acid, and ±0.010 kcal/mole for the water. The uncertainty given is the square root of the sum of the squares of the individual uncertainties.

The heat of vaporization at 25 °C was calculated from the data of Wiberg and Sutterlin [3] as 8.17 ± 0.30 kcal/mole. This leads to the following value for the heat of formation of gaseous dimethoxychloroborane;

$$\begin{array}{c} \Delta Hf{}^{\circ}(25{}^{\circ}C)=-747.89\pm 2.18\;\text{kj}/\text{mole}\\ \;=-178.75\pm 0.52\;\text{kcal}/\text{mole}. \end{array}$$

## 5. Conclusions

**Figure 3:**
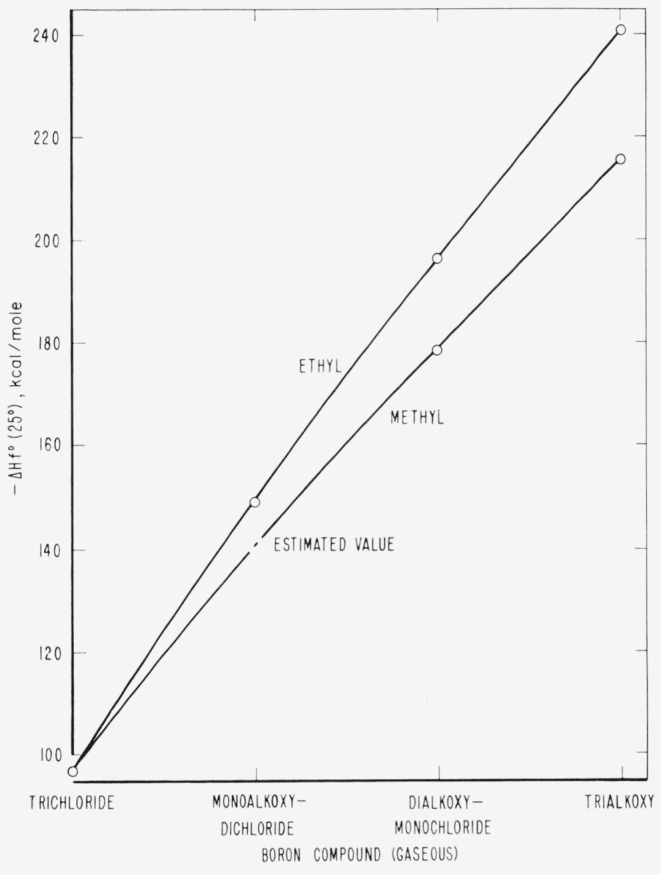
Heats of formation of gaseous alkoxy derivatives of boron trichloride.

Since no value for the heat of formation of dimethoxychloroborane has been reported previously, we can only compare our value with those for other compounds in the same family; i.e., the methoxy and ethoxy derivatives of boron trichloride. These values are given in table 3 and plotted in figure 3. The differences between the ethoxy and the methoxy substitutions are reasonably consistent. One-third of the difference between the values for the trialkoxy derivatives is 8.4 kcal/mole, and one-half of the difference between the values for the dialkoxy derivatives is 8.8 kcal/mole. The difference between the values for the monoalkoxy derivatives is therefore taken as 8.6 kcal/mole. This yields 141.0 kcal/mole for the heat of formation at 25 °C of gaseous methoxydichloroborane.

## Figures and Tables

**Table 1:** Data for the dimethoxychloroborane hydrolysis experiments

Expt. no.	*E_a_*	Δ*Rc*	Wt. of sample	HCl titrated	H_3_BO_3_ titrated	Sample no.	Cl	B	Methyl borate	*q*_MB_	−Δ*H* (26.8 °C)
											
	*j/ohm*	*Ohm*	*g*	*Mole*	*Mole*		*wt. %* (Theor., 32.72)	*wt. %* (Theor., 9.99)	*Mole*	*j*	*kj/mole*
1	21, 200.6	0.023544	0.59815	0.005187	0.005520	10	30.75	9.99	0.000333	5.99	95.08
2	21, 224.2	.040068	1.04074	.008906	.009572	6	30.34	9.95	.000666	11.99	94.14
3	21, 229.7	.034277	0.88547	.007502	.008182	7	30.04	10.00	.000680	12.24	95. 37
5	21, 232.5	.039942	1.05143	.008847	.009640	4–5	29.83	9.92	.000793	14.28	94.25
6	21, 238.6	.030793	0.78938	.006817	.007174	8	30.62	9.83	.000357	6.43	94.99
	
Mean											94.77
Standard deviation of the mean											±0.24

**Table 2:** Data for the boric acid solution experiments

Experiment	Δ*Rc*	−Q*	H_3_BO_3_	Δ*H* (25 °C)
				
	*Ohm*	*j*	*Mole*	*kj/mole*
1	−0.011518	243.95	0.011223	21.737
2	−.013709	290.36	.013133	22.109
3	−.015882	336.38	.015347	21.918
4	−.014360	304.14	.013830	21.991

Mean				21.939
Standard deviation of the mean				±0.078

* Electrical energy equivalent=21,180 j/ohm.

**Table 3:** Heats of formation of some of the gaseous alkoxy derivatives of boron trichloride

Compound	−Δ*Hf*° (25 °C) (gas)
	
	*kcal/mole*
BCl_3_	97.11 [14]
(CH_3_O)BCl_2_	(141.0)*
(CH_3_O)_2_BCl	178.8 ±0.5
(CH_3_O)_3_B	215.7 [1]
(C_2_H_5_O)BCl_2_	149.6 [2]
(C_2_H_5_O)_2_BCl	196.4 [2]
(C_2_H_5_O)_3_B	240.8 [1]

* The value in parentheses is an estimate.

## Footnotes

1 Figures in brackets indicate the literature references at the end of this paper.

2 Pure Substances Section of the Chemistry Division.

3 Analytical Chemistry Section of the Chemistry Division.

4 Trademark.

5 The helium atmosphere was used only because of its availability at the time; a nitrogen atmosphere would have eliminated the small errors resulting from exchange of gases through the Mylar film.

6 Smisko and Mason [101 found no significant change in the heat of dilution between 500 and 5000 moles of water per mole of boric acid; therefore, we assume the value given in equation (2) is the same as for the dilution, 3000 H_2_O per mole of H_3_BO_3_.
